# Savanna in equatorial Borneo during the late Pleistocene

**DOI:** 10.1038/s41598-019-42670-4

**Published:** 2019-04-25

**Authors:** Christopher M. Wurster, Hamdi Rifai, Bin Zhou, Jordahna Haig, Michael I. Bird

**Affiliations:** 1 College of Science and Engineering, James Cook University, Cairns, Queensland 4870 Australia; 2 ARC Centre of Excellence for Australian Biodiversity and Heritage, James Cook University, Cairns, Queensland 4870 Australia; 3 Centre of Tropical Environmental and Sustainability Sciences, James Cook University, Cairns, Queensland 4870 Australia; 4 Department of Physics Faculty of Mathematics and Natural Sciences, Universitas Negeri Padang, Padang, 25131 Indonesia; 5 Key Laboratory of Surficial Geochemistry (Ministry of Education), School of Earth Sciences and Engineering, Nanjing University, Nanjing, China

**Keywords:** Palaeoclimate, Geochemistry

## Abstract

Equatorial Southeast Asia is a key region for global climate change. Here, the Indo-Pacific Warm Pool (IPWP) is a critical driver of atmospheric convection that plays a dominant role in global atmospheric circulation. However, fluctuating sea-levels during the Pleistocene produced the most drastic land-sea area changes on Earth, with the now-drowned continent of Sundaland being exposed as a contiguous landmass for most of the past 2 million years. How vegetation responded to changes in rainfall that resulted from changing shelf exposure and glacial boundary conditions in Sundaland remains poorly understood. Here we use the stable carbon isotope composition (*δ*^13^C) of bat guano and High Molecular Weight *n*-alkanes, from Saleh Cave in southern Borneo to demonstrate that open vegetation existed during much the past 40,000 yrs BP. This location is at the southern equatorial end of a hypothesized ‘savanna corridor’ and the results provide the strongest evidence yet for its existence. The corridor would have operated as a barrier to east-west dispersal of rainforest species, and a conduit for north-south dispersal of savanna species at times of lowered sea level, explaining many modern biogeographic patterns. The Saleh Cave record also exhibits a strong correspondence with insolation and sea surface temperatures of the IPWP, suggesting a strong sensitivity of vegetation to tropical climate change on glacial/interglacial timeframes.

## Introduction

The Indo-Pacific Warm Pool is the largest reservoir of warm water on Earth^1^. Sea surface temperatures (SSTs) exceed 28 °C, and the IPWP plays a critical role in global climate^1,2^. Seasonally, climate is governed by the migration of the Inter-Tropical Convergence Zone (ITCZ), with both the East Asian monsoon (EAM) and the Australian-Indonesian monsoon (AIM) resulting in deep atmospheric convection and high rainfall across the central and northern parts of the region^3,4^. This climate leads to generally everwet conditions and lowland dipterocarp rainforest across most of Borneo today^5^ with tropical grass relative abundance encompassing less than 0.1 for the vast majority of the island^6^. However, abrupt and strong inter-annual variability in SSTs and salinities in the IPWP are associated with ENSO (El Niño-Southern Oscillation) variability that can bring periods of drought^5,7^.

**Figure 1:**
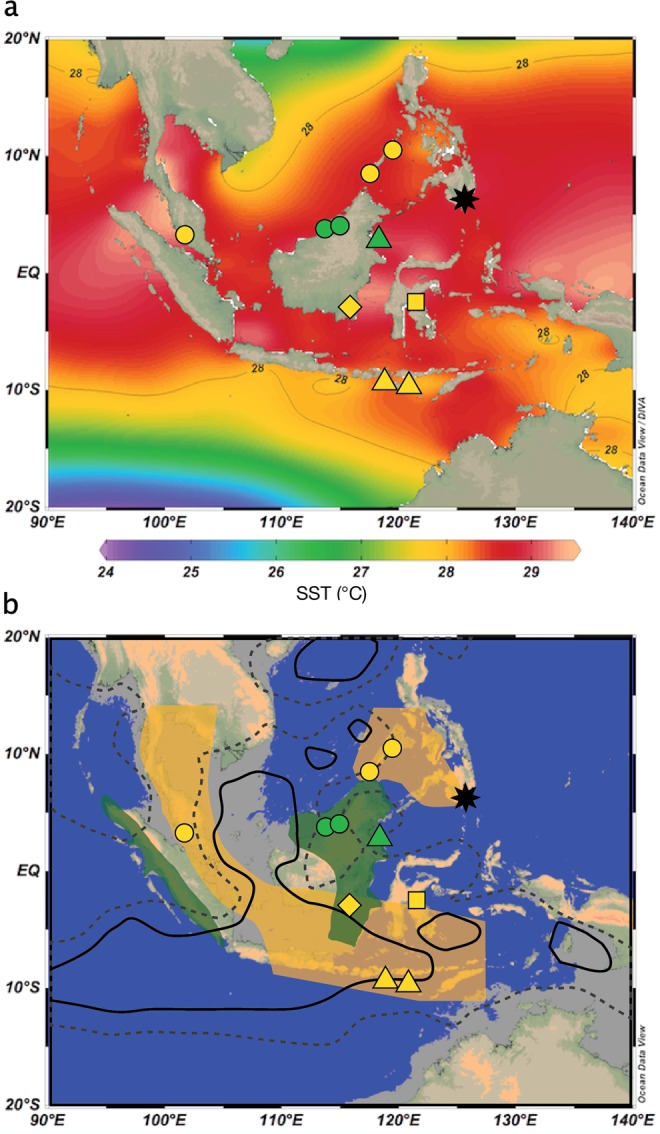
Current and projected LGM conditions surrounding equatorial Southeast Asia. Diamond showing location of Saleh cave (3.0322°S, 115.9839°E) used for palaeoenvironmental reconstruction. Other cave locations (dots), lake sediments (square), offshore terrestrial (triangles), and Sea Surface Temperature (star) records are indicated. Yellow symbols indicate open vegetation and green symbols indicate closed forest were interpreted for the LGM. (**a**) Modern mean annual Sea Surface Temperatures^43^ showing annual averaged 28 °C contours defining the Indo-Pacific Warm Pool. (**b**) 100 m isobaths indicating approximate shoreline of the Last Glacial Maximum. Also shown are proposed glacial savanna and forest refugia^24^, −4 mm/day (solid) and −2 mm/day (dash) contours for annual mean 21 k-0 k precipitation difference for the HadCM3M2 –Ocean-Atmosphere experiment^38^. Figure was constructed using Ocean Data View^44^.

During the Last Glacial Period (LGP, c. 110-11.7 kyr ago), reduced global sea level exposed the continental shelf from south of Thailand to Sumatra, Java, and Borneo, revealing the contiguous continent of Sundaland that reached its maximum land area extent during the Last Glacial Maximum (LGM, 26.5-19 kyr ago^8^) (Fig. 1). Such a large change in land/sea area severely impacted the IPWP by reducing its size^9^ while SSTs 2–4 °C lower than today in the IPWP region would have also served to reduce atmospheric convection^10,11^. It has been proposed that a reduction of rainfall would lead to the development of a ‘savanna corridor’ running north to south/southeast from what is today Peninsula Malaysia across to southern Borneo through the Java Sea^12–14^ (Fig. 1). Whether or not open vegetation existed on Sundaland, especially in Borneo, is the subject of intense debate, with significant implications for its biogeography, conservation, LGP carbon storage, and the understanding of early human dispersals through the region^14,15^.

Vegetation models in general simulate tropical rainforest across Sundaland during the LGM, particularly in central Sundaland^16–18^. Species distribution models also suggested that rich dipterocarp forests were maintained during the LGM in central Sundaland^19^, although a few vegetation models have simulated a possible savanna corridor^18,20^. Vegetation simulations are driven ultimately by climate models that simulate a range of ‘dry’ to ‘wet’ conditions during the LGM for Sundaland^2,21,22^. The limited amount of proxy data in the region has enabled the selective interpretation of sites in order to deduce either drier conditions and open vegetation^2^, or wet conditions and the continued presence of tropical rainforest^21^ during the LGM.

Southern Borneo is ideally placed to test the savanna corridor hypothesis. A few well resolved records in the northern part of Borneo indicate everwet conditions were maintained during the LGP^4,23,24^, the southern part of Borneo has produced only limited and equivocal data. Evidence that southern Borneo was everwet and served as a glacial rainforest refugia, or seasonally dry during the LGM have been summarized elsewhere^13,14^. A few poorly dated or low resolution pollen records from Kalimantan displayed a ‘graminae’ phase during the LGM that have been interpreted as floating mats of herbaceous vegetation, or as representing more seasonal climatic conditions than today^25,26^, while an offshore biomarker record found no change between LGM and Holocene phenol ratios suggesting the maintenance of rainforest^27^.

Herein, we examine vegetation change over the past 40,000 cal yrs BP using *δ*^13^C values of guano and High Molecular Weight (HMW) *n*-alkanes extracted from a 3 m thick guano deposit in South Kalimantan, Indonesia (Saleh Cave, 3.03220°S, 115.98386°E). Faeces (guano) can accumulate in deposits several meters thick in Southeast Asia, providing sedimentary proxy records of environmental change amenable to radiocarbon dating^28^. Fresh guano is composed dominantly of finely comminuted insect cuticles that are subsequently broken down by bacteria and fungi. These sediments contain multi-proxy information from a variety of sources, but *δ*^13^C values are arguably the most powerful proxy for past environments that can be derived from tropical guano records^24,29^.

Insect abundance is largely determined by available vegetation^30^, and insect *δ*^13^C values are determined by diet with little fractionation^31^. Communities of bats and swiftlets generally forage within a 15 km range of the roost^32^ ensuring that a local to regional vegetation signal is captured in the guano deposit^33,34^. HMW *n*-alkanes represent molecular records from insect cuticles, both from assimilation of plant derived *n*-alkanes, and from *de novo* synthesis^35,36^ (see supplemental text). In lowland tropical locations, grasses utilize the C_4_ photosynthetic pathway, whereas trees use the C_3_ pathway. The different enzymatic pathways used to fix CO_2_ results in *δ*^13^C values of C_4_ plants (−9 to −16‰) and their insect hosts that are substantially different than those of C_3_ plants (−19 to −34‰)^37^. Hence, both guano and HMW *n*-alkanes *δ*^13^C values indicate the abundance of local to regional C_4_ biomass. As seasonality of precipitation is a strong predictor of vegetation in the Sundaic region^4^, changing relative abundance of C_4_ vegetation indicates changing precipitation regimes.

## Tropical grass expansion in southern Borneo during the LGP

**Figure 2:**
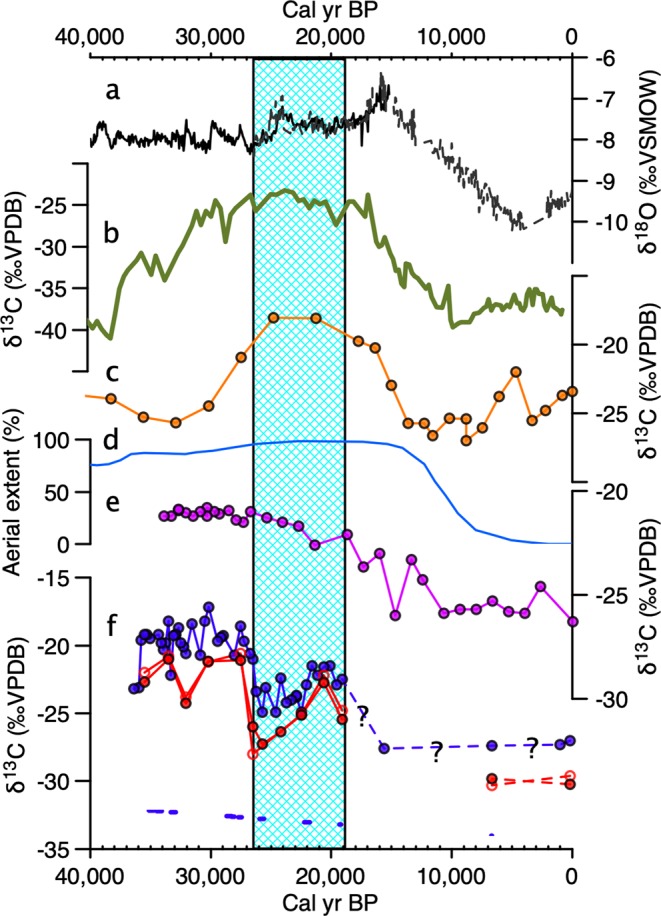
Comparison of guano *δ*^13^C records to regional records of palaeoclimate from key regions in and around Sundaland. (**a**) Cave stalagmite *δ*^18^O records from Borneo^23,45^. (**b**) *δ*^13^C values from fatty acids from Lake Towuti, Sulawesi^39^. (**c**) Guano *δ*^13^C record from Palawan^23^. (**d**) Aereal extent of Sunda shelf exposure^38^. (**e**) Guano *δ*^13^C record from Peninsular Malaysia^23^. (**f**) Guano (blue) and *n*-alkane records from Saleh Cave (red, C_27_: closed, C_29_: open). Calibrated age ranges (2σ) from radiocarbon measurements are plotted. Highlighted area indicates the Last Glacial Maximum.

**Figure 3:**
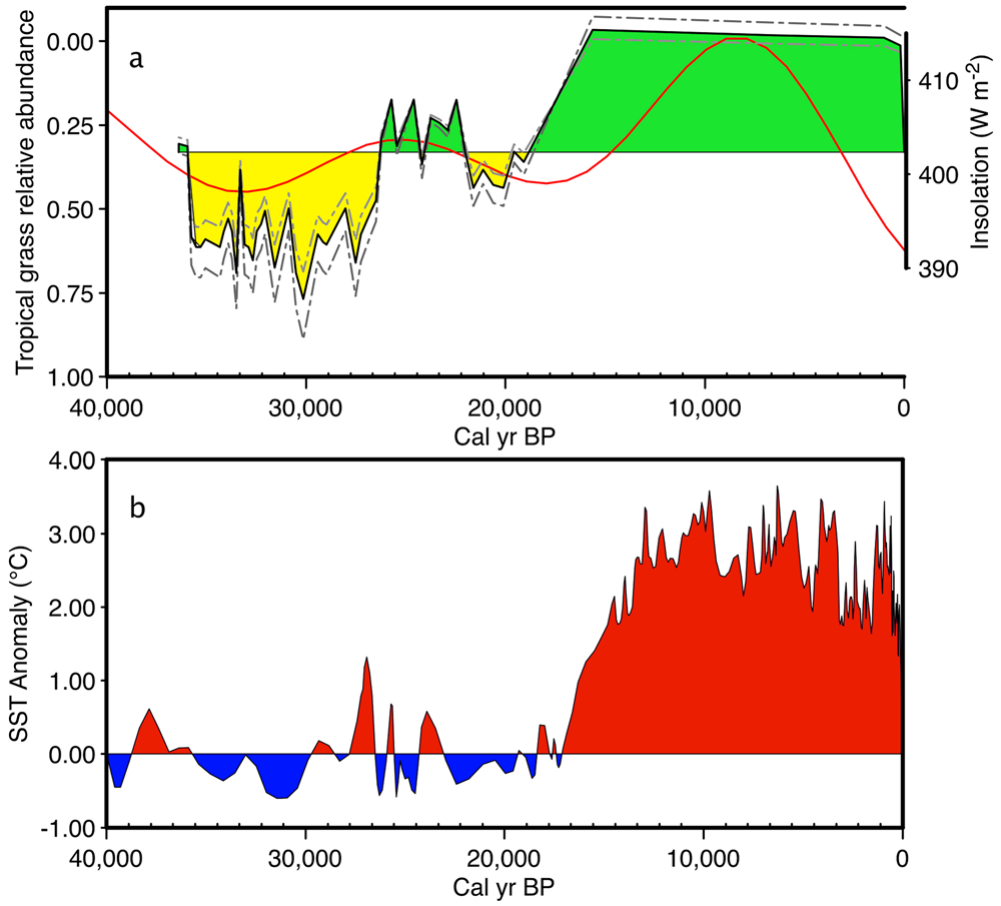
Comparison of C_4_ ratio estimated from the Saleh Cave *δ*^13^C guano record with Sea Surface Temperatures of the IPWP. (**a**) Tropical grass relative abundance as estimated by an empirical equation and a mass balance model (see supplementary information). Data is filled yellow below 0.22, which corresponds with the *δ*^13^C value that contains an unambiguous tropical grass component (see supplementary information), and green when above that corresponding ratio. Also plotted is July insolation at 0° ^46^ a, Sea Surface Temperature is plotted using a 6.7 low pass butter-worth filter for Celebes sea derived from Mg/Ca of *Globigerinoides ruber* foraminifera^41^. Sea Surface Temperature is determined relative to average glacial temperatures from that core and filled red when above, and blue when below.

We use guano and HMW *n*-alkane *δ*^13^C values in a guano deposit from Saleh Cave, South Kalimantan, Indonesia, to infer past variations in vegetation. Ten radiocarbon dates on guano provide a chronology for these records (Supplementary text, Supplementary Table 1, Supplementary Fig. 1). Guano *δ*^13^C values at Saleh Cave ranged between −17.2 and −27.3‰, a total range of 10.1‰ (Fig. 2). The most salient feature of the guano *δ*^13^C profile is that the highest values (−17.2‰ to −22.5‰) unambiguously indicate that a major expansion of tropical grasses occurred between 35 and 26.5 cal kyr BP (Fig. 3). At 26.5 cal kyr BP, *δ*^13^C values decreased rapidly to values between −23.4 and −24.9‰. A second, expansion of more open environments occurred during the LGM, with *δ*^13^C values as high as −21.5‰. Holocene *δ*^13^C values (<−27‰) are the most negative in our record, reflecting wet conditions that enabled development of a closed tropical rainforest environment that is consistent with observed modern vegetation.

HMW *n*-alkanes are represented in the Saleh record by the dominant *n*-alkanes C_27_ and C_29_ with *δ*^13^C values that show a similar pattern when compared against *δ*^13^C values of guano (r = 0.73, r = 0.90, p < 0.01 for C_27_ and C_29_ *δ*^13^C values compared against *δ*^13^C guano, respectively). The largest range is evident in C_27_ *δ*^13^C values, (9.7‰), although a similar range (9.0‰) was measured for C_29_. Coeval patterns among *n*-alkanes and guano *δ*^13^C values occurred that included highest values across all proxies between 35 and 26.5 cal kyr BP, a notable decrease at ~26 cal kyr BP, and increased values during the LGM before decreasing again during the Holocene (Fig. 2).

High *δ*^13^C values from the Saleh Cave guano provide unequivocal evidence that relatively open and dry environments were present in southern Borneo during the Late Pleistocene (Figs 2, 3). δ^13^C values of guano as high as −17.2‰ are the most positive yet measured for guano in Southeast Asia, implying up to 70–89% C_4_ production (Fig. 3). Other regional records show interesting similarities and differences with the Saleh cave record (Fig. 2). Records from the continent of Sundaland such as Batu guano in peninsular Malaysia^24^, Saleh guano (this study), and to some extent δ^18^O values of speleothems in northern Borneo (Carolin *et al*., 2013) show a broad trend of dry climate well before the LGM, and no distinct difference during the LGM and late MIS 3. Notably, the Sunda shelf was largely exposed well before the LGM, and may have significantly impacted climate on these locations (Fig. 2d). By contrast, records from locations disconnected from the exposed landmass, such as Palawan and Sulawesi, indicate a drying from at least 35 kyr BP with maximal drying during the LGM. Regional differences might be expected in this complex region. Annual mean 21k-0k precipitation difference for the HadCM3M2 –Ocean-Atmosphere experiment^38^ indicated a complex pattern of rainfall anomalies (Fig. 1b).

In general, speleothem *δ*^18^O records from caves in northern Borneo also suggested a drier LGP relative to the Holocene, although a guano record in Sarawak^24^ and fatty acid *δ*^13^C values from an offshore record^4^ indicated the presence of closed-canopy vegetation suggesting any reductions in precipitation were modest. Other regional records indicate a dry LGP with more open vegetation^4,39,40^. The stable carbon isotope composition of guano from northern and southern Palawan showed distinctly more positive *δ*^13^C values during the LGM identified in both insect cuticles and HMW *n*-alkanes^24^. This is similar to lake sediment fatty acid *δ*^13^C values from Sulawesi that also indicated a dry period extending from 30,000 to c. 17,000 yr BP^39,40^. Moreover, fatty acid *δ*^13^C values indicate that an expansion in tropical grasses and herbs occurred during the LGM on the shelf exposed near the lesser Sunda islands south of Sulawesi^4^. A significant period of drying is inferred from the cave guano values for much of the LGP, with maximal expression prior to the LGM, in our study area. This finding is similar to that found at a continental site at the north western side of the purported savanna corridor^24^. The Saleh Cave guano record provides the first unambiguous terrestrial evidence from Borneo, at the equatorial end of the purported ‘savanna corridor’, that distinctly open-canopy vegetation occurred during the LGP. This record, in combination with those in northern Sundaland provides the strongest evidence yet of the existence of a ‘savanna corridor’^14^.

## Vegetation response to insolation and IPWP SST

An additional feature of the Saleh stable isotope records is that δ^13^C profiles vary in concert with insolation at the equator during boreal summer (Fig. 3). At this time, precessional forcing is strong, and indicates a sensitivity with vegetation. Prior to the early Holocene peak, July insolation is lowest at 33 kyr BP and highest at 25 kyr BP, which closely tracks vegetation change at Saleh cave as inferred from δ^13^C values. Vegetation sensitivity to insolation surrounding Saleh cave could be translated rapidly via vegetation feedbacks and SSTs in the IPWP as recorded in the Celebes Sea^41^. The Celebes Sea record also bears strong similarities to the savanna record at Saleh, where relatively cool (warm) SST in the Celebes Sea is closely associated with dry (wet) conditions in South Kalimantan, Indonesia (Fig. 3). This includes a period of cool SSTs from at least 40 to 27.5 cal kyr BP when the highest *δ*^13^C values of guano and HMW *n*-alkanes occurred in the Saleh Cave record. An abrupt increase in SST occurred from c. 27.7 until 23 cal kyr, coeval with wetter conditions inferred from the Saleh Cave record (Fig. 3). Relatively cool SSTs returned during the LGM, coincident with a further expansion of open vegetation with a significant tropical grass component at Saleh Cave. Early Holocene SSTs gradually increased to the currently warm IPWP regime, and, although compromised by a loss of sample resolution (see methods), the Saleh record demonstrates that closed rainforest canopy was established during the Holocene. We find that increased SST in the IPWP, even under glacial conditions, is associated with a reduction in C_4_ biomass in southern Borneo (Fig. 3). Although, analogies based on present-day ENSO dynamics fail to describe tropical climate change in the region during the LGM due to shelf exposure, it appears that there is a strong linkage between SSTs of the IPWP and vegetation during the LGP, not unlike those of today, with El Niño associated with reduced convection, particularly in southern Borneo as compared with the north^42^.

The Saleh Cave record adds to an emerging picture of significant climate heterogeneity in the region, with high sensitivity to glacial-interglacial forcing. A north-south savanna corridor from peninsular Malaysia, through central Sundaland during glacial periods would represent a ‘barrier’ for rainforest specialists as it imposed restrictions on east-west dispersal, thereby increasing local endemism in rainforest refugia east and west of the corridor^14^. In contrast, the corridor provided a north-south ‘bridge’ facilitating dispersal for organisms that favor open habitats. The legacy of this corridor is evident in the genetic diversity and species distributions observed across island Southeast Asia today^13,14^. In addition, the savanna corridor may have facilitated rapid early human dispersal through the region^14^.

## Methods

### Study Site, Sampling, and Chronology

Saleh Cave (3.03220º S, 115.98386º E, 48 m) is located in a tropical karst tower along the eastern edge of South Kalimantan, Indonesia, approximately 25 (200) km from the modern (LGM) coast (Fig. 1). It is a small shelter extending approximately 500 m into the karst with unidentified insectivorous bats presently inhabiting the cave, and two open chambers each containing a separate and large guano deposits. The nearest deposit to the entrance was very wet and pocketed by drip holes in the surface. We collected from the drier and larger chamber further from the entrance. Surface and ancient ‘rock guano’ (at depth) were collected from a pit excavated through accumulated guano to bedrock at 304 cm depth. Exposed sediments were sampled at 3–5 cm intervals, adjusted where necessary to ensure that sample intervals did not cross stratigraphic boundaries. Samples were irradiated to sterilize, then kept frozen until freeze-dried.

A chronology was established by AMS ^14^C measurements on solvent extracted guano using acid/base/acid treatment (see Supplementary Table 1). A few samples were problematic, and these were repeated by isolating pyrogenic carbon using hydrogen pyrolysis and submitted for radiocarbon measurement (see Supplementary information). An age model was constructed using Bacon 2.2 which employs Bayesian analysis to reconstruct accumulation rates for depositional sequences, through combining radiocarbon dates with prior information such as accumulation rate and its variability. The output is a probability distribution of calibrated dates with depth (Blaauw and Christen, 2011). Calendar year as a function of depth was plotted using weighted mean age output (Supplementary Fig. 1). Radiocarbon measurements indicate that guano miners removed much of the Holocene guano. Modern guano contribution to surface samples (down to 20 cm) was estimated via carbon abundances, and guano age to 25 cm was estimated via mass balance between contemporary guano and ancient guano, hence an apparent low accumulation rate above 20 cm.

### Determination of Carbon Isotope Composition

Briefly, guano samples were crushed using a mortar and pestle, and lipids were removed by sonicating in 2:1 (v/v) dichloromethane/ methanol 3 times, followed with one rinse in methanol. Solvent extracted guano (SEG) was then washed in 1 M NaOH at 100 °C for 30 minutes, neutralised using DI-water and subsequently washed for 3 hrs in 2 M HCl, and neutralized before freeze-drying again (SEG-BA). Carbon isotope composition was determined using an elemental analyzer (ECS 4010 CHNSO Analyzer; Costech Analytical Technologies INC, Valencia, CA, USA) fitted with a Costech Zero Blank Autosampler coupled via a ConFloIV to a Thermo Scientific Delta V^PLUS^ using Continuous-Flow Isotope Ratio Mass Spectrometry (EA-IRMS) at the Advanced Analytical Centre, James Cook University, Cairns. Calibration curves for elemental abundances were determined for two in-house standards (Chitin and Taipan) and USGS-40 (L-glutamic acid) within the same analysis sequence, and reproducibility was better than ±5% of the value. *δ*^13^C values are reported as per mil (‰) deviations from the VPDB reference scale, using the same reference materials as for elemental abundances. Precisions (S.D.) on internal standards were better than ±0.1‰.

*n*-alkane fractions were extracted from guano using a CEM MARS (Microwave accelerated reaction system) with 2:1 DCM:MeOH (v/v). Filtered samples were further cleaned using silica gel flash chromatography. The compound-specific carbon isotope composition of *n-*alkanes was measured with a Thermo Scientific Trace GC instrument connected to a Thermo Scientific Delta Plus XP isotope ratio mass spectrometer (GC-IRMS), equipped with a DB-5 MS column (30 m × 0.25 mm × 0.25 μm). The injector was set at 300 °C. The GC oven temperature was held at 50 °C for 1 min, then set to 220 °C at 10 °C/min (held 2 min), and then to 300 °C (2 °C/min), and finally to 310 °C (10 °C/min, held for 20 min). He was used as carrier gas (1.4 ml/min). The temperature of the combustion oven was 950 °C. Instrumental performance was verified before and after each sample run using an *n*-alkane standard mixture with known *δ*^13^C values (*n*-C16–*n*-C30, Indiana University). The relative standard deviation for reference material was better than ±0.5‰, based on a minimum of two analyses. Results are reported as per mil (‰) deviations from the VPDB reference scale.

## Data Availability

The datasets generated during and/or analysed during the current study are available from the corresponding author on reasonable request.

## Supplementary information

Supplementary information
