# Author Correction: Savanna in equatorial Borneo during the late Pleistocene

**DOI:** 10.1038/s41598-020-60958-8

**Published:** 2020-03-03

**Authors:** Christopher M. Wurster, Hamdi Rifai, Bin Zhou, Jordahna Haig, Michael I. Bird

**Affiliations:** 1 College of Science and Engineering, James Cook University, Cairns, Queensland 4870 Australia; 2 ARC Centre of Excellence for Australian Biodiversity and Heritage, James Cook University, Cairns, Queensland 4870 Australia; 3 Centre of Tropical Environmental and Sustainability Sciences, James Cook University, Cairns, Queensland 4870 Australia; 4 Department of Physics Faculty of Mathematics and Natural Sciences, Universitas Negeri Padang, Padang, 25131 Indonesia; 5 Key Laboratory of Surficial Geochemistry (Ministry of Education), School of Earth Sciences and Engineering, Nanjing University, Nanjing, China

Correction to: *Scientific Reports* 10.1038/s41598-019-42670-4, published online 25 April 2019

In this Article, the references for Figure 2c, 2d, and 2e are incorrect. As a result, the legend for Figure 2:

“Comparison of guano δ^13^C records to regional records of palaeoclimate from key regions in and around Sundaland. (a) Cave stalagmite δ18O records from Borneo^23,45^. (b) δ^13^C values from fatty acids from Lake Towuti, Sulawesi^39^. (c) Guano δ^13^C record from Palawan^23^. (d) Aereal extent of Sunda shelf exposure^38^. (e) Guano δ^13^C record from Peninsular Malaysia^23^. (f) Guano (blue) and n-alkane records from Saleh Cave (red, C27: closed, C29: open). Calibrated age ranges (2σ) from radiocarbon measurements are plotted. Highlighted area indicates the Last Glacial Maximum.”

should read:

“Comparison of guano δ^13^C records to regional records of palaeoclimate from key regions in and around Sundaland. (a) Cave stalagmite δ18O records from Borneo^23,45^. (b) δ^13^C values from fatty acids from Lake Towuti, Sulawesi^39^. (c) Guano δ^13^C record from Palawan^24^. (d) Aereal extent of Sunda shelf exposure^39^. (e) Guano δ^13^C record from Peninsular Malaysia^24^. (f) Guano (blue) and n-alkane records from Saleh Cave (red, C27: closed, C29: open). Calibrated age ranges (2σ) from radiocarbon measurements are plotted. Highlighted area indicates the Last Glacial Maximum.”

