# Supplementary material for: Savanna in equatorial Borneo during the late Pleistocene
Source: Sci Rep. 2019 Apr 25;9:6392. doi: 10.1038/s41598-019-42670-4 (PMC6483998; doi:10.1038/s41598-019-42670-4)
Supplement: Supplementary file 1 — Supplementary information [file 41598_2019_42670_MOESM1_ESM.pdf]

Savanna in equatorial Borneo during the late Pleistocene

Christopher M. Wurster*[1,2,3], Hamdi Rifai[4], Bin Zhou[5], Jordahna Haig[1,2,3], and Michael I. Bird[1,2,3]

[1]*College of Science and Engineering, James Cook University, Cairns, Queensland, 4870, Australia.*

[2] *ARC Centre of Excellence for Australian Biodiversity and Heritage, James Cook University, Cairns, Queensland, 4870, Australia.*

[3]*Centre of Tropical Environmental and Sustainability Sciences, James Cook University, Cairns, Queensland, 4870, Australia.*

[4]*Department of Physics Faculty of Mathematics and Natural Sciences, Universitas Negeri Padang, Padang 25131, Indonesia.*

[5]*Key Laboratory of Surficial Geochemistry (Ministry of Education), School of Earth Sciences and Engineering, Nanjing University, Nanjing, China.*

**correspondence to Christopher.wurster@jcu.edu.au*

Supplementary information

The age model for the Saleh Cave guano deposit

A total of 10 samples were submitted for radiocarbon dates (Supplementary Table 1). Radiocarbon dates were measured at the Waikato Radiocarbon Dating Laboratory and the Australian Nuclear Science and Technology Organisation (ANSTO). Radiocarbon measurements were calibrated to calendar years using SHCal[1]. An age model for each profile was constructed using Bacon 2.2, and a probability distribution is determined as a function of depth (Supplementary Fig. 1).

Two samples were identified as outliers by Bacon 2.2, and were repeated by isolating pyrogenic carbon using hydrogen pyrolysis (hypy), a new technique for determining reliable radiocarbon measurements[2,3]. Briefly, samples were immersed in 2M HNO_3 for 3 hours followed by 30% peroxide overnight to remove inorganic and labile carbon, then loaded with a Mo catalyst using an aqueous/methanol (1:1) solution of ammonium dioxydithiomolybdate [$(NH_4)_2MoO_2S_2$]. Catalyst weight was ~5 % sample weight for all samples to give a nominal loading of 1% Mo. Catalyst loaded samples were then lyophilized and placed in the hypy reactor, pressurized with hydrogen to 15 GPa with a sweep gas flow of 5 L min^{-1}, then heated using a pre-programmed temperature profile. We used the recommended temperature program previously optimized for pyrogenic carbon quantification where samples are initially heated at rate of 300 °C min^{-1} to 250 °C, then heated at a rate of 8 °C min^{-1} until the final hold temperature of 550 °C for 2 min[2].

Table S1. Radiocarbon dates from the Saleh Cave guano profile

Sample	Laboratory code	Extraction material[1]	% Modern Carbon	^{14}C age	Calibrated Cal yr BP (min-mid-max, (95.4%)[2]
10-15 cm	40986	SEG-ABA	50.30±0.10	5,516±21	6208-6280-6313
27-30 cm	40340	SEG-ABA	14.3+0.1	15,640±48	18722-18843-18965
50-55 cm	OZQ028	SEG-ABA	10.95±0.11	17,770±180	20932-21450-21917
95-100 cm	OZQ031	SEG-ABA	5.07±0.11	23,950±170	27668-27968-28380
95-100 cm	OZW062	PyC	7.36±0.12	20,960±140	24756-25249-25630
145-150 cm	40987	SEG-ABA	5.3±0.10	23,643±126	27500-27719-27932
195-200 cm	40341	SEG-ABA	5.9±0.1	22,772±104	26690-27098-27376
195-200 cm	OZW064	PyC	2.85±0.04	28,590±120	31944-32584-33041
255-260 cm	40988	SEG-ABA	2.5±0.1	29,655±216	33395-33786-34170
290-295 cm	41594	SEG-ABA	2.3±0.1	30,458±249	33958-34402-34845

[1]CExtraction method for Solvent Extracted-Acid-Base-Acid (SEG-BA) and Pyrogenic Carbon (PyC), are described in the main and supplementary texts, respectively. [2]Calibration output from OXCAL 4.3[4] using SHCal13[1].

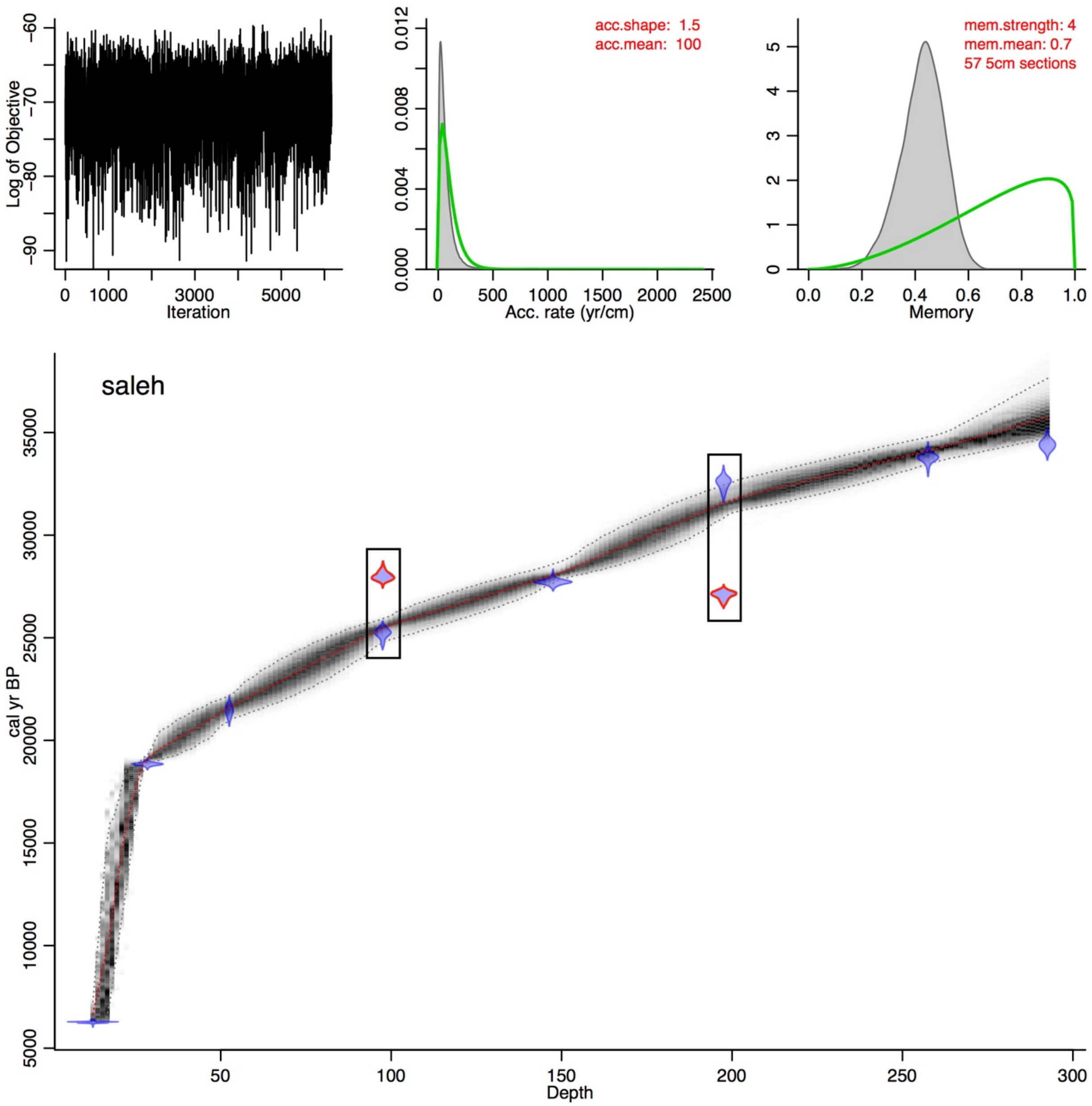

Figure S1. Age-depth relationship Bacon[5] output for Saleh Cave radiocarbon measurements showing probability distribution of ages as a function of depth. Boxes surround two repeated measurements, where the Guano SEG (red) was determined to be an outlier and repeated by isolating pyrogenic carbon.

Estimation of tropical grass biomass contribution.

In order to estimate tropical grass (e.g., C_4) biomass contribution, two independent estimations were assessed in order to provide a possible range in values: (1) Empirically derived C_4 plant estimations determined from a study on insectivorous bat guano[6] and (2) assuming a simple mass balance model.

Empirically derived estimates

An empirically derived equation for bat guano from the southwest US has been published[6], and subsequently modified to assume that sites with less than 25 mm precipitation/year have no contribution of C4 biomass (Eq 1)[7].

$$\delta^{13}C_{guano} = 10.8 \bullet (C_4 \text{ relative abundance}) - 26.8 \quad (1)$$

Mass balance

The abundance of C_4 biomass was determined using a simple mass balance model where:

$$\delta^{13}C_{guano} = f_{C4} \bullet (\delta^{13}C_{C4} + \varepsilon_4) + (1 - f_{C4}) \bullet (\delta^{13}C_{C3} + \varepsilon_3) \quad (2)$$

where $\delta^{13}C_{guano}$ is the $\delta^{13}C$ value of guano, f_{C4} is the proportion of C_4 biomass, $\delta^{13}C_{C4}$ is the average $\delta^{13}C$ value of C_4 biomass, $\delta^{13}C_{C3}$ is the average $\delta^{13}C$ value of C_3 biomass and ε is the fractionation between dietary plant biomass and insect cuticles (which may be different between C_4 and C_3 vegetation). C_4 biomass can be estimated to be -12.5‰ and C_3 can be estimated to be -27.5‰[7,8]. Under different atmospheric $\delta^{13}CO_2$ conditions, it is possible to compensate for changed plant endmember values by simply adding the difference to the insect cuticle $\delta^{13}C$ value. However, this parameter deviated by no more than 0.5‰ over the last 22,000 years, so we did not incorporate this change in our calculation. We take ε values from insects cultured on C_3 or C_4 biomass[9], where $\varepsilon_3 = 0.8$, and $\varepsilon_4 = 0$.

Changes in $\delta^{13}C$ values of forest end-members.

The stable carbon isotope composition of forests varies with the type of forest present[10]. A more seasonal climate might have resulted in tropical seasonal forests or tropical deciduous forests replacing tropical rainforest. Baseline $\delta^{13}C$ values are approximately 2 ‰ more positive for tropical seasonal forests relative to rainforest, and tropical deciduous forests are 3.8 ‰ higher than tropical rainforest[10]. Notably, $\delta^{13}C$ values from Saleh cave are above baseline $\delta^{13}C$ values for even tropical deciduous seasonal forests, unambiguously indicating that tropical grasses were a substantial part of the environments of southern Borneo during most of the past 40,000 years and that the hydroclimate was significantly drier and/or more seasonal (Supplementary Fig. 2).

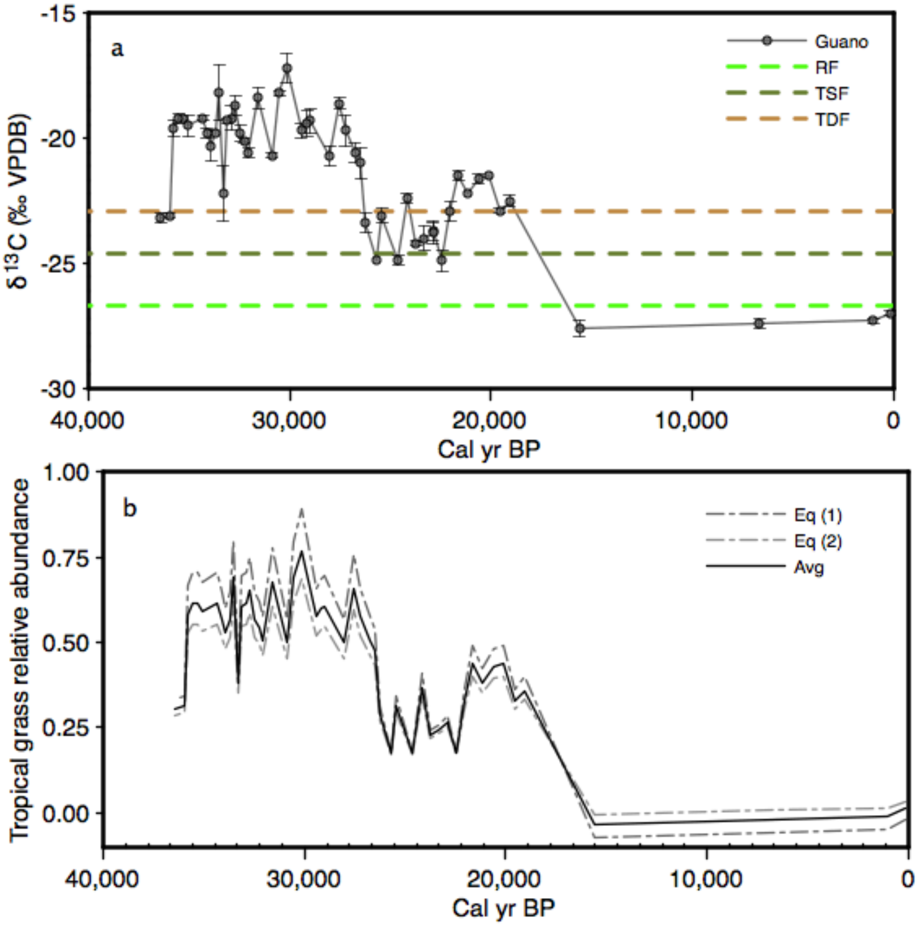

Figure 2. Environmental reconstruction surrounding Saleh cave for the past 40,000 years. a, Variation in carbon stable isotope composition. Also noted are lines indicating 100 % C_3 vegetation if rainforest (RF), Tropical Seasonal Forest (TSF), or Tropical Deciduous Forest (TDF) are considered. Guano $\delta^{13}C$ values above respective lines indicate C_4 was present. b, Estimation of Tropical grass relative abundance via 2 independent methods (see supplementary text for more information).

High molecular weight *n*-alkanes in guano.

Guano deposits are composed of the undigested components of the bat communities diet, which is largely composed of insect cuticles[11]. Insects produce a variety of hydrocarbons, including linear *n*-alkanes, alkenes, methyl branched alkanes, and alkadienes[12]. Although high molecular weight *n*-alkanes with a strong odd over even preference are almost exclusively used as a molecular biomarker of vascular plants in sedimentary records[13], insects also produce these compounds with a strong odd over even preference[14,15]. A detailed investigation of the hydrocarbons in bat guano determined that although insects almost exclusively produced

branched alkanes, high-molecular weight *n*-alkanes (e.g., C27, C29), were directly assimilated into insect cuticles via plants and synthesized by the insects[15]. In fact, it was estimated that odd high-molecular weight *n*-alkanes were assimilated vs synthesized at a ratio of approximately 0.3, whereas even *n*-alkanes were mostly assimilated directly from plants at a ratio of ~0.7. Nonetheless, $\delta^{13}C$ values of high-molecular weight *n*-alkanes are a record of local vegetation, as both synthesized and assimilated compounds are directly reflective of the insects diet[14]. The strong covariation with guano $\delta^{13}C$ values yields confidence that both proxies yield a reliable signal without diagenetic interference.

Supplementary References

1. Hogg, A. G. *et al.* SHCal13 Southern Hemisphere Calibration, 0–50,000 Years cal BP. *Radiocarbon* **55,** 1889–1903 (2013).
2. Ascough, P. L. *et al.* Hydropyrolysis as a new tool for radiocarbon pre-treatment and the quantification of black carbon. *Quat. Geochronol.* **4,** 140–147 (2009).
3. Bird, M. I. *et al.* The efficiency of charcoal decontamination for radiocarbon dating by three pre-treatments – ABOX, ABA and hypy. *Quat. Geochronol.* **22,** 25–32 (2014).
4. Ramsey, C. B., Scott, E. M. & van der Plicht, J. Calibration for Archaeological and Environmental Terrestrial Samples in the Time Range 26–50 ka cal BP. *Radiocarbon* **55,** 2021–2027 (2013).
5. Blaauw, M. & Christen, J. A. Flexible paleoclimate age-depth models using an autoregressive gamma process. *Bayesian Anal.* **6,** 457–474 (2011).
6. Wurster, C. M., McFarlane, D. A. & Bird, M. I. Spatial and temporal expression of vegetation and atmospheric variability from stable carbon and nitrogen isotope analysis of bat guano in the southern United States. *Geochim. Cosmochim. Acta* **71,** 3302–3310 (2007).
7. Wurster, C. M. *et al.* Forest contraction in north equatorial Southeast Asia during the Last Glacial Period. *Proc. Natl. Acad. Sci.* **107,** 15508–15511 (2010).

8. Randerson, J. T. *et al.* Fire emissions from C_3 and C_4 vegetation and their influence on interannual variability of atmospheric CO_2 and $\delta^{13}CO_2$. *Glob. Biogeochem. Cycles* **19,** GB2019 (2005).

9. Gratton, C. & Forbes, A. E. Changes in δ^{13}C stable isotopes in multiple tissues of insect predators fed isotopically distinct prey. *Oecologia* **147,** 615–624 (2006).

10. Diefendorf, A. F., Mueller, K. E., Wing, S. L., Koch, P. L. & Freeman, K. H. Global patterns in leaf ^{13}C discrimination and implications for studies of past and future climate. *Proc. Natl. Acad. Sci.* **107,** 5738–5743 (2010).

11. Wurster, C. M., Munksgaard, N., Zwart, C. & Bird, M. The biogeochemistry of insectivorous cave guano: a case study from insular Southeast Asia. *Biogeochemistry* **124,** 163–175 (2015).

12. Drijfhout, F., Kather, R. & Martin, S. J. The role of cuticular hydrocarbons in insects. in *Behavioral and Chemical Ecology* (eds. Zhang, W. & Liu, H.) 91–114 (Nova Science Publishers, 2010).

13. Eglinton, T. I. & Eglinton, G. Molecular proxies for paleoclimatology. *Earth Planet. Sci. Lett.* **275,** 1–16 (2008).

14. Des Marais, D. J., Mitchell, J., Meinschein, W. & Hayes, JM. The carbon isotope biogeochemistry of the individual hydrocarbons in bat guano and the ecology of the insectivorous bats in the regions of Carlsbad, New Mexico. *Geochim. Cosmochim. Acta* **44,** 2075–2086 (1980).

15. Chikaraishi, Y., Kaneko, M. & Ohkouchi, N. Stable hydrogen and carbon isotopic compositions of long-chain (C21–C33) *n*-alkanes and *n*-alkenes in insects. *Geochim. Cosmochim. Acta* **95,** 53–62 (2012).
